# Clinical characteristics of Parkinson’s disease in the outpatient clinic of a regional hospital in Peru

**DOI:** 10.17843/rpmesp.2025.424.15105

**Published:** 2025-12-13

**Authors:** José J. Centeno-Arispe, Carlos A. Hurtado-González, Alan P. Murillo-Salas, Isabel P. Camargo-Salazar, Karlo J. Lizarraga-Mendoza

**Affiliations:** 1 Hospital Regional Honorio Delgado Espinoza, Arequipa, Perú. Hospital Regional Honorio Delgado Espinoza Arequipa Perú; 2 Universidad Católica de Santa María, Arequipa, Peru. Universidad Católica de Santa María Universidad Católica de Santa María Arequipa Peru; 3 Universidad Cooperativa de Colombia, Cali, Valle del Cauca, Colombia. Universidad Cooperativa de Colombia Universidad Cooperativa de Colombia Cali Valle del Cauca Colombia; 4 Universidad Nacional de San Agustín, Arequipa, Peru. Universidad Nacional de San Agustín Universidad Nacional de San Agustín Arequipa Peru; 5 Universidad de Rochester, Rochester, Nueva York, Estados Unidos de Norteamérica. University of Rochester Universidad de Rochester Rochester Nueva York USA

**Keywords:** Parkinson´s Disease, Cognitive Dysfunction, Depression, Anxiety

## Abstract

Parkinson’s Disease (PD) is the fastest-growing neurodegenerative disorder worldwide. The clinical characteristics of adults with PD in a regional referral hospital in Arequipa were determined through a descriptive and cross-sectional study conducted between June 2023 and January 2025. Sixty adults with PD were included (20 women), and the average age was 64.4 years. The most frequent motor phenotype was the tremor-dominant type (73.3%). All received tablets with 250 mg of levodopa and 25 mg of carbidopa. Half took between 250 and 750 mg of levodopa daily, and 21% presented with dyskinesias. The most frequent non-motor symptoms were anxiety (93.2%), depression (88.6%), and mild cognitive impairment (>60%). These findings highlight the opportunity to optimize the treatment of motor and non-motor symptoms with pharmacological and non-pharmacological therapies focused on improving the quality of life of PD patients residing in the Arequipa region.

## INTRODUCTION

Parkinson’s Disease (PD) is the fastest-growing neurodegenerative disorder worldwide and is characterized by motor and non-motor symptoms ^1^. Its diagnosis is based on clinical criteria, as no specific biomarkers are currently available ^2^. International studies indicate that it affects men over 60 years of age more frequently, with tremor being the predominant symptom ^3^. In Peru, local research reveals that PD is more frequent in older adults, with a predominance in males, and manifests mainly with motor symptoms such as bradykinesia and tremor. Among the most common non-motor symptoms are depression and insomnia ^4^^-^^5^.

Specific epidemiological studies on PD in southern Peru have not been published, including the Arequipa region, which is home to 1,631,136 inhabitants, making it the second most populated department in Peru. The Hospital Regional Honorio Delgado Espinoza (HRHDE), a tertiary referral facility in southern Peru, maintains a registry of patients with PD, developed following its participation in training programs in neurology and movement disorders ^6^^-^^10^. This registry allows for studies on the frequency and clinical characteristics of PD in this region. The objective of the present study was to determine the clinical characteristics of adults with PD treated in the outpatient clinic of the HRHDE in Arequipa, Peru.

**KEY MESSAGES**

Study motivation: No studies have been published on the clinical characteristics of patients with PD in the Arequipa region.

Main findings: Patients with PD presented a high percentage of non-motor symptoms such as depression, anxiety, and cognitive impairment. The levodopa dose was relatively low compared to that used in other populations worldwide.

Public health implications: These findings could support the implementation of regional programs focused on optimizing the comprehensive treatment of motor and non-motor symptoms through the use of pharmacological and non-pharmacological therapies.

## THE STUDY

### Design and population

A descriptive cross-sectional study was conducted. Out of a total of 102 individuals with a diagnosis of PD referred to the hospital, 60 patients who voluntarily agreed to participate and were treated in the neurology outpatient clinic between June 2023 and January 2025 were included. Two patients with severe psychiatric disorders were excluded.

### Selection, sample, and sampling

The sampling type was non-probabilistic by convenience, including new and follow-up patients who agreed to a neurological evaluation performed during a visit separate from their regular care. All demographic and clinical information was collected directly through a research interview with the participant.

### Variables

The diagnosis of PD was made according to the United Kingdom Brain Bank criteria ^11^. Other variables considered were: age (in years), sex, age at onset of motor symptoms, age at diagnosis, duration and type of onset (tremor-dominant or rigid/akinetic), laterality of the initial primary symptom, stage according to the Hoehn and Yahr (H&Y) classification, non-motor symptoms according to Part I of the official Spanish translation of the MDS-Unified Parkinson’s Disease Rating Scale (MDS-UPDRS), motor function according to Part III of the MDS-UPDRS scale, cognitive function according to the Montreal Cognitive Assessment (MoCA) and MoCA S1-2 ^12^, doses of carbidopa/levodopa and other antiparkinsonian medications, and dyskinesias as motor complications associated with levodopa use (supplementary material).

### Statistical analysis

A descriptive analysis was performed using measures of central tendency and dispersion for numerical variables, calculating the mean and range (minimum - maximum). Frequencies and percentages were calculated for nominal variables. All analyses were performed using Microsoft Excel and IBM SPSS Statistics version 30.0 software.

### Ethical criteria

Informed consent was obtained from all participants. The study was conducted with the approval of the Ethics Committee of the Universidad de San Agustín de Arequipa (Format 3/No. 018 CIEI/UI/FM/UNSA).

## FINDINGS

The analysis was performed on a cohort of 60 patients with PD. The mean age was 64.4 years (range: 42−92), with a predominance of the male sex (66.7%). A total of 51.7% had between 7 and 11 years of schooling, while 13.8% had no formal education. The mean age of motor symptom onset was 58.2 years, with a mean disease duration of 6.2 years. Five cases (8%) were identified with onset before age 40. The tremor-dominant motor phenotype was observed in 73.3% of the patients, with right laterality in 56.7%. The mean score on the UPDRS III was 55.8 in the OFF state and 28.8 in the ON state. Of the 38 patients evaluated in the ON state, eight (21%) presented with dyskinesias, most with more than 10 years of disease progression. Most were in stage 2 of the H&Y classification (53.3%) (Table 1).

**Table 1:** Demographic and clinical characteristics of patients with Parkinson’s disease at the Hospital Regional Honorio Delgado Espinoza, Arequipa, Peru.

Characteristics		Frequency	%
Age in years (N=60)		64.4 (42-92)	-
Sex (N=60)			
	Male	40	66.7
	Female	20	33.3
Years of formal education (N=58)			
	No education	8	13.8
	1 to 6	17	29.3
	7 to 11	30	51.7
	12 or more	3	5.2
Age in years at symptom onset (N=60)		58.2 (28-85)	-
Disease duration in years (N=60)		6.2 (0-19)	-
Delay in PD diagnosis in years			-
	All patients (N=60)	1.1 (0-12)	-
	Patients with onset before age 40 (N=5)	3.6 (0-12)	
	Patients with onset after age 40 (N=55)	0.9 (0-10)	
Laterality of initial symptom (N=60)			
	Right	34	56.7
	Left	23	38.3
	Bilateral	3	5.0
Predominant motor phenotype (N=60)			
	Tremor-dominant	44	73.3
	Rigid/Akinetic	16	26.7
Receives levodopa treatment (tablets with 250 mg levodopa and 25 mg carbidopa)			
	No	5	8.3
	Yes	55	91.7
Levodopa dose (mg/day) (N=55)			
	< 250	10	18.2
	251 - 500	18	32.7
	501 - 750	12	21.8
	751 - 1000	6	10.9
	> 1000	9	16.4
MDS-UPDRS a III in ON state (N=38)		28.8 (3-81)	-
MDS-UPDRS a III in OFF state (N=22)		55.8 (18-108)	-
Hoehn and Yahr (N=60)			
	1	9	15.0
	2	32	53.3
	3	14	23.3
	4	4	6.7
	5	1	1.7
Dyskinesias b			
	With dyskinesias	8	21.1
	Without dyskinesias	30	78.9
MoCA c (N=48)		19.1 (10-27)	-
MoCA S1-2 d (N=48)		19.4 (11-27)	-

a MDS-Unified Parkinson’s Disease Rating Scale.

b Based on the 38 patients evaluated in the ON state in whom the presence of dyskinesias was observed.

c Montreal Cognitive Assessment. Cut-off point for cognitive impairment <26.

d Considers the influence of schooling on the evaluation by adding two points for schooling <8 years and one point for schooling between 8 and 12 years. Cut-off point for cognitive impairment <21.

**Figure 1:**
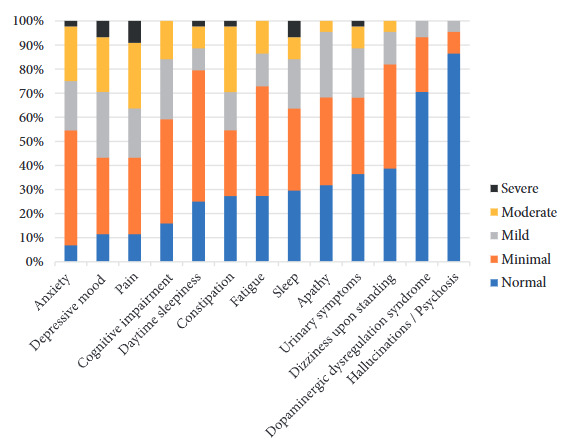
Non-motor symptoms according to the MDS-UPDRS Part I scale in patients with Parkinson’s disease at Honorio Delgado Espinoza Regional Hospital, Arequipa, Peru.

Regarding non-motor symptoms, 93.2% presented with anxiety symptoms, 88.6% with depressive mood, 88.7% with pain, and 84.1% with cognitive impairment, manifesting as frequent forgetfulness or memory problems (Figure 1). Cognitive impairment was identified in more than 60% of the evaluated patients (87.5% using the MoCA and 62.5% using the MoCA s1-2).

**Figure 2:**
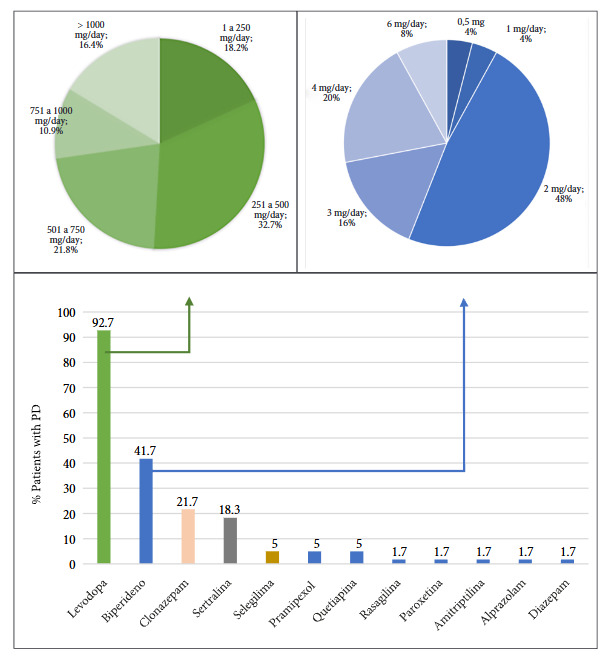
Usual medication in patients with Parkinson’s disease at Honorio Delgado Espinoza Regional Hos-pital, Arequipa, Peru.

The analysis of pharmacological treatment for motor symptoms revealed that 92.7% of patients received carbidopa/levodopa. Additionally, 41.7% used biperiden, primarily for managing resistant tremor or in cases of levodopa intolerance. Regarding the treatment of non-motor symptoms, 21.7% of patients used clonazepam for insomnia or anxiety, and 18.3% received sertraline for the treatment of depression (Figure 2).

## DISCUSSION

It was found that 8% of patients began the disease before age 40. The tremor-dominant phenotype was the most frequent (73.3%), and more than 80% of patients presented with non-motor symptoms. Furthermore, 92.7% of patients received carbidopa/levodopa.

The higher prevalence of PD in males, with a mean age over 60 years, coincides with reports from other studies conducted in hospital centers within the country ^4^. The mean age of motor symptom onset was 58.2 years (range 28−85 years), and five patients began the disease before age 40. This proportion is within the range described for cases of early-onset PD, which ranges from 5 to 10% of total cases ^13^. This finding is relevant as an average diagnosis delay of 3.6 years was observed in patients with onset before age 40, compared to 0.9 years in those with later onset. This suggests that in the region, the perception persists that PD is exclusively a disease of old age.

Regarding motor symptoms, the most frequent phenotype was tremor-dominant. Dyskinesia was present in 21% of patients examined in the ON state. The frequency of motor complications usually ranges between 40% and 50% after 4-6 years of treatment and can reach up to 90% after eight years ^14^. In our cohort, the mean disease duration was 6.2 years, and most patients with dyskinesias had more than 10 years of disease.

The lower frequency of dyskinesias observed compared to other studies could be due to the use of lower doses of carbidopa/levodopa. This might be related to tolerability, considering that globally, most have access to formulations of 25 mg carbidopa and 100 mg levodopa (1:4 ratio), whereas in the present study, all patients received formulations of 25 mg carbidopa and 250 mg levodopa (1:10 ratio), which increases the risk of adverse effects and would not allow for dose escalation ^15^. Additionally, the fear of patients and healthcare providers regarding increasing the levodopa dose could be another contributing factor ^16^, especially in contexts where there is limited access to therapies for managing motor complications.

It is important to note that recent research has confirmed that the levodopa dose should be adjusted to improve the patient’s quality of life and not for the purpose of avoiding motor complications, which tend to appear regardless of the duration of use or the administered dose ^17^. While access to treatments for motor complications increases in our environment, future studies could investigate the use of other therapies that might be more accessible to reduce the severity of motor complications.

Another possible consequence of using relatively low doses of levodopa is the persistence of tremor, which would be even more relevant in patients with the tremor-dominant phenotype who have limited access to other therapies. As a possible consequence, 41.7% of patients were receiving biperiden, a figure higher than the 24% reported in other populations ^9^^,^^18^. The use of biperiden has been associated with adverse effects, including cognitive impairment; therefore, it is not recommended in current therapeutic guidelines.

It is essential to conduct neuropsychological studies to expand cognitive evaluations in clinical practice. These evaluations would be useful in low-income countries with little access to advanced treatments for levodopa-resistant tremor, such as deep brain stimulation. It is recommended to implement prospective studies that more accurately analyze the progression of cognitive impairment and its relationship with treatment. Meanwhile, the levodopa dose could be increased using formulations that improve its tolerability. If this is not possible, we must carefully balance the possible risks and benefits of using medications such as biperiden.

Regarding non-motor symptoms, a high frequency was observed, exceeding 80%, including anxiety and depression. These figures are high compared to similar studies using the MDS-UPDRS Part I, where cognitive impairment (71.9%), pain (65.7%), fatigue (61.7%), and depression (57.2%) were found as predominant symptoms ^(^^19^. Likewise, it was observed that approximately 20% of patients received pharmacological treatment such as clonazepam or sertraline, and none received non-pharmacological therapies to address these manifestations. Cognitive impairment was present in 87.5% of patients according to the MoCA scale and 62.5% with the MoCA S1-2. These figures are higher than other studies where cognitive impairment varies between 24% and 45% ^20^.

Non-motor symptoms are frequent in PD patients and significantly affect their quality of life. The findings of this study highlight the need to include mental health and cognitive evaluations, including neuropsychological tests that allow for the characterization of the phenotype and the patient’s baseline state, with the aim of evaluating the response of non-motor symptoms to possible pharmacological and non-pharmacological therapies, ideally as part of comprehensive treatment programs.

One of the main limitations of the study was the sample size. Although most patients referred to the neurology outpatient clinic with a PD diagnosis were registered, it was not possible to evaluate everyone because some did not complete their care or were referred to other specialties due to comorbidities. Another important limitation was the exclusive use of the MoCA screening instrument for cognitive evaluation, without applying a complete neuropsychological battery. This was due to the lack of neuropsychologists trained in evaluating patients with neurodegenerative diseases in the hospital. Likewise, Part IV of the MDS-UPDRS was not applied, which would have allowed for a more precise characterization of the presence and severity of motor complications associated with levodopa treatment.

In conclusion, the findings of this study show characteristics similar to other populations, except for the greater diagnostic delay in patients under 40 years of age, the use of relatively low doses of levodopa from a single formulation, and the higher frequency of non-motor symptoms without adequate diagnosis or treatment, especially anxiety, depression, and cognitive impairment. These findings can be explained in part by limited access to other levodopa formulations and advanced therapies such as deep brain stimulation. These findings highlight the opportunity to optimize the treatment of motor and non-motor symptoms with pharmacological and non-pharmacological therapies, focused on improving the quality of life of PD patients living in the Arequipa region, ideally as part of comprehensive treatment programs that include physical therapy, psychotherapy, and functional neuro-rehabilitation plans.
