# Supplementary material for: Clinical characteristics of Parkinson’s disease in the outpatient clinic of a regional hospital in Peru
Source: Rev Peru Med Exp Salud Publica. 2025 Dec 13;42(4):430–5. doi: 10.17843/rpmesp.2025.424.15105 (PMC12879983; doi:10.17843/rpmesp.2025.424.15105)
Supplement: Supplementary material. — Available in the electronic version of the RPMESP. [file rpmesp-42-04-15105-s001.docx]

TABLA 1 (Material suplementario)

**9.1 Operacionalización de variables de interés**

| **VARIABLES** | **DEFINICIÓN OPERACIONAL** | **CRITERIOS** | **UNIDAD DE MEDIDA** | **TIPO DE VARIABLE** |
| --- | --- | --- | --- | --- |
| **Edad** | Edad actual del participante al momento de la evaluación. | Fecha de nacimiento | Años cumplidos | Numérica  Continua |
| **Sexo** | Identificación del sexo biológico según características del participante | Características sexuales secundarias | Masculino  Femenino | Nominal |
| **Nivel de instrucción** | Años de estudio formal del participante | Según respuesta | Sin instrucción  De 1 a 6 años  De 7 a 11 años  De 12 a más | Nominal |
| **Edad de inicio de síntomas motores** | Edad en la cual el participante manifiesta haber iniciado los síntomas | Según encuesta | Años | Razón |
| **Edad de diagnóstico de enfermedad** | Edad en la cual el participante manifiesta haber sido diagnosticado de la enfermedad de Parkinson | Según encuesta | Años | Razón |
| **Edad de inicio de tratamiento con levodopa** | Edad en la cual el participante manifiesta haber iniciado el uso de levodopa | Según encuesta | Años | Razón |
| **Lateralidad** | Lado del cuerpo que el participante manifiesta haber iniciado los síntomas | Según encuesta | Derecha  Izquierda | Nominal |
| **Tipo** | Tipo de síntoma que el participante manifiesta como más evidente | Según encuesta | Tremolante  Rígido-akinético | Nominal |
| **Discinesias** | Movimientos hipercinéticos relacionados al uso de levodopa | Según encuesta | Sí  No | Nominal |
| **Tratamiento con Levodopa** | Cantidad de medicación referida por el paciente | Según encuesta | <250mg  251mg a 500mg  501mg a 750mg  751mg a 1000mg  >1000mg | Ordinal |
| **Tratamiento con Pramipexol** | Cantidad de medicación referida por el paciente | Según encuesta | Dosis actual | Ordinal |
| **Tratamiento con Biperideno** | Cantidad de medicación referida por el paciente | Según encuesta | Dosis actual | Ordinal |
| **Tratamiento con Rasagilina** | Cantidad de medicación referida por el paciente | Según encuesta | Dosis actual | Ordinal |
| **Tratamiento con Selegilina** | Cantidad de medicación referida por el paciente | Según encuesta | Dosis actual | Ordinal |
| **Tratamiento con antidepresivos** | Cantidad de medicación referida por el paciente | Según encuesta | Dosis actual | Ordinal |
| **Tratamiento con ansiolíticos** | Cantidad de medicación referida por el paciente | Según encuesta | Dosis actual | Ordinal |
| **MDS- UPDRS Parte I** | Evaluación neurológica realizada al paciente | Según escala | Normal:0  Mínimo: 1  Leve: 2  Moderado: 3  Grave: 4 | Ordinal |
| **MDS- UPDRS Parte III** | Entrevista estructurada realizada al paciente | Según escala | Normal:0  Mínimo:1  Leve: 2  Moderado:3  Grave: 4 | Ordinal |
| **Hoehn & Yahr** | Evaluación neurológica realizada al paciente | Según escala | Asintomático: 0  Compromiso motor unilateral:1  Afectación bilateral: 2  Inestabilidad postural:3  Discapacidad grave: 4  Silla de ruedas o cama:5 | Ordinal |
| **MoCA** | Entrevista y evaluación del paciente | Según escala | Normal: 26 puntos a más  Trastorno cognitivo: 0-25 puntos | Ordinal |
| **MoCA S1-2** | Entrevista y evaluación del paciente | Según escala | Normal: 21 puntos a más  Trastorno cognitivo: 0-20 puntos | Ordinal |
