# Supplementary material for: Clinical characteristics of Parkinson’s disease in the outpatient clinic of a regional hospital in Peru
Source: Rev Peru Med Exp Salud Publica. 2025 Dec 13;42(4):430–5. doi: 10.17843/rpmesp.2025.424.15105 (PMC12879983; doi:10.17843/rpmesp.2025.424.15105)
Supplement: Translation

# Translation: Características clínicas de la enfermedad de Parkinson en consulta externa de un hospital regional en Perú

**Authors:** José J. Centeno-Arispe, Carlos A. Hurtado-González, Alan P. Murillo-Salas, Isabel P. Camargo-Salazar, Karlo J. Lizarraga-Mendoza

**Affiliations:** 1 Hospital Regional Honorio Delgado Espinoza, Arequipa, Perú.; 2 Universidad Católica de Santa María, Arequipa, Peru.; 3 Universidad Cooperativa de Colombia, Cali, Valle del Cauca, Colombia.; 4 Universidad Nacional de San Agustín, Arequipa, Perú.; 5 University of Rochester, Rochester, New York, United States of America.

**Keywords:** Enfermedad de Parkinson, Disfunción Cognitiva, Depresión, Ansiedad

## Abstract

La Enfermedad de Parkinson (EP) es el trastorno neurodegenerativo de más rápido crecimiento mundial. Se determinaron las características clínicas de adultos con EP en un hospital de referencia regional en Arequipa, mediante un estudio descriptivo y transversal realizado entre junio de 2023 y enero de 2025. Se incluyeron 60 adultos con EP (20 mujeres), la edad promedio fue de 64,4 años. El fenotipo motor más frecuente fue el tremolante (73,3%). Todos recibían tabletas con 250 mg de levodopa y 25 mg de carbidopa. La mitad tomaba entre 250 y 750 mg de levodopa al día y el 21% presentaba discinesias. Los síntomas no motores más frecuentes fueron la ansiedad (93,2%), la depresión (88,6%) y el deterioro cognitivo leve (>60%). Estos hallazgos resaltan la oportunidad de optimizar el tratamiento de síntomas motores y no motores con terapias farmacológicas y no farmacológicas enfocadas en mejorar la calidad de vida de los pacientes con EP residentes en la región Arequipa.

## INTRODUCCIÓN

La Enfermedad de Parkinson (EP) es el trastorno neurodegenerativo de más rápido crecimiento a nivel mundial y se caracteriza por síntomas motores y no motores ^1^. Su diagnóstico se basa en criterios clínicos, ya que actualmente no se dispone de biomarcadores específicos ^2^. Estudios internacionales indican que afecta con mayor frecuencia a hombres mayores de 60 años, siendo el temblor el síntoma predominante ^3^. En el Perú, investigaciones locales revelan que la EP es más frecuente en adultos mayores, con predominio en varones y se manifiesta principalmente con síntomas motores como bradicinesia y temblor. Entre los síntomas no motores más comunes se encuentran la depresión y el insomnio ^4^^-^^5^.

No se han publicado estudios epidemiológicos específicos sobre la EP en el sur del Perú, incluida la región de Arequipa que alberga a 1 631 136 habitantes, constituyéndose así en el segundo departamento más poblado del Perú. El Hospital Regional Honorio Delgado Espinoza (HRHDE), establecimiento de referencia de tercer nivel en el sur del Perú, cuenta con un registro de pacientes con EP, desarrollado tras su participación en programas de capacitación en neurología y trastornos del movimiento ^6^^-^^10^. Este registro permite la realización de estudios sobre la frecuencia y características clínicas de la EP en esta región. El objetivo del presente estudio fue determinar las características clínicas de adultos con EP atendidos en consulta externa del HRHDE en Arequipa, Perú.

**MENSAJE CLAVE**

Motivación del estudio. No se han publicado estudios sobre de las características clínicas de los pacientes con EP en la región Arequipa.

Principales hallazgos. Los pacientes con EP presentaron un elevado porcentaje de síntomas no motores como depresión, ansiedad y deterioro cognitivo. La dosis de levodopa fue relativamente baja en comparación con la utilizada en otras poblaciones a nivel mundial.

Implicancias en salud pública. Estos hallazgos podrían respaldar la implementación de programas regionales enfocados en optimizar el tratamiento integral de los síntomas motores y no motores, mediante el uso de terapias farmacológicas y no farmacológicas.

## EL ESTUDIO

### Diseño y población

Se realizó un estudio descriptivo transversal. De un total de 102 personas con diagnóstico de EP referidas al hospital, se incluyeron a 60 pacientes que aceptaron participar voluntariamente y que fueron atendidos en la consulta externa del servicio de neurología entre junio del 2023 y enero del 2025. Se excluyeron dos pacientes que presentaron trastornos psiquiátricos severos.

### Selección, muestra y muestreo

El tipo de muestreo fue no probabilístico, por conveniencia, que incluyó a pacientes nuevos y continuadores que accedieron a una evaluación neurológica, realizada en una consulta diferente a su atención regular. Toda la información demográfica y clínica fue recolectada directamente mediante una entrevista de investigación con el participante.

### Variables

El diagnóstico de EP se realizó de acuerdo con los criterios del Banco de Cerebros del Reino Unido ^11^. Se consideró otras variables: la edad (en años), sexo, edad de inicio de los síntomas motores, edad de diagnóstico, duración y tipo de inicio (tremolante o rígido/acinético), lateralidad del síntoma principal inicial, estadio según la clasificación de Hoehn y Yahr (HyY), síntomas no motores según la Parte I de la traducción oficial al Español de la escala MDS-Unified Parkinson Disease Rating Scale (MDS-UPDRS), función motora según la Parte III de la escala MDS-UPDRS, función cognitiva de acuerdo a la escala Montreal Cognitive Assessment (MoCA) y MoCA S1-2 ^12^, dosis de carbidopa/levodopa y de otros medicamentos antiparkinsonianos y discinesias como complicaciones motoras asociadas al uso de levodopa (material suplementario).

### Análisis estadístico

Se realizó un análisis descriptivo mediante medidas de tendencia central y dispersión para las variables numéricas, calculando la media y el rango (mínimo - máximo). Para las variables nominales se calcularon frecuencias y porcentajes. Todos los análisis se realizaron con Microsoft Excel y el software IBM SPSS Statistics versión 30.0.

### Criterios éticos

Se obtuvo el consentimiento informado de todos los participantes. El estudio se realizó con la aprobación del Comité de Ética de la Universidad de San Agustín de Arequipa (Formato 3/Nº 018 CIEI/UI/FM/UNSA).

## HALLAZGOS

El análisis se realizó en una cohorte de 60 pacientes con EP. La edad promedio fue de 64,4 años (rango: 42−92), con predominio del sexo masculino (66,7%). El 51,7% tenía entre 7 y 11 años de estudios, mientras que el 13,8% no tenía escolaridad. La edad promedio de inicio de síntomas motores fue de 58,2 años, con una evolución media de la enfermedad de 6,2 años. Se identificaron cinco casos (8%) con inicio antes de los 40 años. El fenotipo motor tremolante fue observado en el 73,3% de los pacientes, con lateralidad derecha en el 56,7%. El puntaje promedio en la UPDRS III fue 55,8 en estado OFF y 28,8 en estado ON. De los 38 pacientes evaluados en estado ON, ocho (21%) presentaron discinesias, la mayoría con más de 10 años de evolución de la enfermedad. La mayoría se encontraba en estadio 2 de la clasificación HyY (53,3%) (tabla 1).

**Tabla 1:** Características demográficas y clínicas de los pacientes con enfermedad de Parkinson en el Hospital Regional Honorio Delgado Espinoza, Arequipa, Perú.

Características		Frecuencia	%
Edad en años (N=60)		64,4 (42-92)	-
	Sexo (N=60)		
	Masculino	40	66,7
	Femenino	20	33,3
Años de estudios formales (N=58)			
	Sin estudios	8	13,8
	De 1 a 6	17	29,3
	De 7 a 11	30	51,7
	De 12 a más	3	5,2
Edad en años de inicio de síntomas (N=60)		58,2 (28-85)	-
Tiempo de enfermedad en años (N=60)		6,2 (0-19)	-
Retraso en el diagnostico de EP en años			-
	Todos los pacientes (N=60)	1,1 (0-12)	-
	Pacientes que iniciaron antes de los 40 años (N=5)	3,6 (0-12)	
	Pacientes que iniciaron después de los 40 años (N=55)	0,9 (0-10)	
Lateralidad del síntoma inicial (N=60)			
	Derecha	34	56,7
	Izquierda	23	38,3
	Bilateral	3	5,0
Fenotipo motor predominante (N=60)			
	Tremolante	44	73,3
	Rígido/Akinetico	16	26,7
Recibe tratamiento con levodopa (tabletas con 250 mg de levodopa y 25 mg de carbidopa)			
	No	5	8,3
	SÍ	55	91,7
Dosis de levodopa (mg/día) (N=55)			
	< 250	10	18,2
	251 - 500	18	32,7
	501 - 750	12	21,8
	751 - 1000	6	10,9
	> 1000	9	16,4
MDS-UPDRS ^a^ III en estado ON (N=38)		28,8 (3-81)	-
MDS-UPDRS ^a^ III en estado OFF (N=22)		55,8 (18-108)	-
Hoehn and Yahr (N=60)			
	1	9	15,0
	2	32	53,3
	3	14	23,3
	4	4	6,7
	5	1	1,7
Discinesias ^b^			
	Con discinesias	8	21,1
	Sin discinesias	30	78,9
MoCA ^c^ (N=48)		19,1 (10-27)	-
MoCA S1-2 ^d^ (N=48)		19,4 (11-27)	-

a MDS-Unified Parkinson’s Disease Rating Scale

b En base a los 38 pacientes evaluados en estado ON que se objetivo la presencia de discinesias

c Montreal Cognitive Assessment. Punto de corte para deterioro cognitivo <26.

d Considera la influencia de la escolaridad en la evaluación adicionando dos puntos para escolaridad <8 años y un punto para escolaridad entre 8 y 12 años. Punto de corte para deterioro cognitivo <21.

En cuanto a los síntomas no motores, el 93,2% presentó síntomas de ansiedad, el 88,6% de ánimo depresivo, el 88,7% dolor y el 84,1% deterioro cognitivo, manifestándose como olvidos frecuentes o problemas de memoria (figura 1 ). El deterioro cognitivo se identificó en más del 60% de los pacientes evaluados (87,5% usando el MoCA y 62,5% usando el MoCA s1-2).

**Figura 1:** Sintomas no motores según la escala MDS-UPDRS Parte I en pacientes con enfermedad de Par-kinson en el Hospital Regional Honorio Delgado Espinoza, Arequipa, Perú.

El análisis del tratamiento farmacológico para síntomas motores reveló que el 92,7% de los pacientes recibía carbidopa/levodopa. Además, el 41,7% utilizaba biperideno principalmente para el manejo del temblor resistente o en casos de intolerancia a la levodopa. Respecto al tratamiento de síntomas no motores, el 21,7% de los pacientes utilizaba clonazepam para insomnio o ansiedad, y el 18,3% recibía sertralina para el tratamiento de la depresión (figura 2).

**Figura 2:** Medicación habitual en pacientes con enfermedad de Parkinson en el Hospital Regional Honorio Delgado Espinoza, Arequipa, Perú.

## DISCUSIÓN

Se hallo que el 8% de los pacientes inició la enfermedad antes de los 40 años. El fenotipo tremolante fue el más frecuente (73,3%) y más del 80% de los pacientes presento síntomas no motores. Además, el 92,7% de los pacientes recibió carbidopa/levodopa.

La mayor prevalencia de EP en varones, con una edad promedio superior a los 60 años, coincide con lo reportado en otros estudios realizados en centros hospitalarios del país ^4^. La edad promedio de inicio de síntomas motores fue de 58,2 años (rango 28−85 años), y cinco pacientes iniciaron la enfermedad antes de los 40 años. Esta proporción se encuentra en el rango descrito para casos de EP de inicio temprano, que oscila entre el 5 a 10% del total de casos ^13^. Este hallazgo es relevante, ya que se observó un retraso promedio en el diagnóstico de 3,6 años en pacientes con inicio antes de los 40 años, en comparación con 0,9 años en aquellos con inicio posterior a esa edad. Esto sugiere que, en la región, persiste la percepción de que la EP es una enfermedad exclusiva de la tercera edad.

En cuanto a los síntomas motores, el fenotipo más frecuente fue el tremolante. La discinesia se presentó en el 21% de los pacientes examinados en estado ON. La frecuencia de complicaciones motoras suele oscilar entre el 40% y 50% después de 4-6 años de tratamiento, y puede alcanzar hasta el 90% después de ocho años ^14^. En nuestra cohorte el tiempo medio de evolución fue de 6,2 años, y la mayoría de los pacientes con discinesias tenían más de 10 años de enfermedad.

La menor frecuencia de discinesias observada en comparación con otros estudios podría deberse al uso de dosis más bajas de carbidopa/levodopa. Esto podría relacionarse con la tolerabilidad, considerando que a nivel mundial en su mayoría se tiene acceso a formulaciones de carbidopa 25mg y levodopa 100mg (relación 1:4), y en el presente estudio todos los pacientes recibieron formulaciones de carbidopa 25mg y levodopa 250mg (relación 1:10) que aumenta el riesgo de efectos adversos y no permitiría incrementar la dosis ^15^. Adicionalmente, el temor de pacientes y proveedores de salud a aumentar la dosis de levodopa podría ser otro factor contribuyente ^16^, especialmente en contextos donde existe un acceso limitado a terapias para el manejo de las complicaciones motoras.

Es importante señalar que investigaciones recientes han confirmado que la dosis de levodopa debe ajustarse para mejorar la calidad de vida del paciente y no con la finalidad de evitar la aparición de complicaciones motoras, las cuales tienden a presentarse independientemente del tiempo de uso o la dosis administrada ^17^. Mientras aumenta el acceso a los tratamientos para las complicaciones motoras en nuestro medio, estudios futuros podrían investigar el uso de otras terapias que podrían ser más accesibles para disminuir la severidad de las complicaciones motoras.

Otra posible consecuencia de usar dosis relativamente bajas de levodopa es la persistencia del temblor, lo cual sería aún más relevante en pacientes con fenotipo tremolante que tienen acceso limitado a otras terapias. Como posible consecuencia, el 41,7% de pacientes venia recibiendo biperideno, una cifra superior al 24% reportado en otras poblaciones ^9^^,^^18^. El uso de biperideno se ha asociado con efectos adversos, incluyendo deterioro cognitivo, por lo cual no se recomienda en las guías terapéuticas vigentes.

Es fundamental realizar estudios neuropsicológicos para ampliar las evaluaciones cognitivas en la práctica clínica. Estas evaluaciones serían útiles en países con bajos ingresos y poco acceso a tratamientos avanzados para el temblor resistente a levodopa, como la estimulación cerebral profunda. Se recomienda implementar estudios prospectivos que analicen con mayor precisión la progresión del deterioro cognitivo y su relación con el tratamiento. Mientras tanto, se podría aumentar la dosis de levodopa usando formulaciones que mejoren su tolerabilidad. Si esto no es posible, debemos balancear cuidadosamente los posibles riesgos y beneficios del uso de medicamentos como el biperideno.

En cuanto a los síntomas no motores, se observó una elevada frecuencia, superior al 80%, incluyendo ansiedad y depresión. Estas cifras son altas en comparación con estudios similares que utilizaron la MDS-UPDRS Parte I, en donde se encontró como síntoma predominante el deterioro cognitivo (71,9%), dolor (65,7%), fatiga (61,7%) y depresión (57,2%) ^19^. Asimismo, se observó que aproximadamente el 20% de pacientes recibía tratamiento farmacológico como clonazepam o sertralina, y que ninguno recibía terapias no farmacológicas para abordar estas manifestaciones. El deterioro cognitivo se presentó en el 87,5% de pacientes mediante la escala MoCA y 62,5% con el MoCA S1-2. Estas cifras son superiores a otros estudios en donde el deterioro cognitivo varía entre 24% y 45% ^20^.

Los síntomas no motores son frecuentes en pacientes con EP y afectan significativamente su calidad de vida. Los hallazgos de este estudio resaltan la necesidad de incluir evaluaciones de salud mental y cognitiva, incluyendo pruebas neuropsicológicas que permitan caracterizar el fenotipo y el estado basal del paciente, con el objetivo de evaluar la respuesta de los síntomas no motores a posibles terapias farmacológicas y no farmacológicas, idealmente como parte de programas de tratamiento integral.

Una de las principales limitaciones del estudio fue el tamaño de la muestra. Si bien, se logró registrar a la mayor parte de los pacientes referidos al consultorio externo de neurología con el diagnostico de EP, no fue posible evaluar a todos, debido a que algunos no completaron su atención o fueron derivados a otras especialidades debido a sus comorbilidades. Otra limitación importante fue el uso exclusivo del instrumento de tamizaje MoCA para la evaluación cognitiva, sin la aplicación de una batería neuropsicológica completa. Esto se debió a la falta de neuropsicólogos capacitados en la evaluación de pacientes con enfermedades neurodegenerativas en el hospital. Asimismo, no se aplicó la Parte IV de la MDS-UPDRS, lo que habría permitido una caracterización más precisa de la presencia y severidad de las complicaciones motoras asociadas al tratamiento con levodopa.

En conclusión, los hallazgos de este estudio muestran características similares a otras poblaciones, salvo por el mayor retraso en el diagnostico para pacientes menores de 40 años, el uso de dosis relativamente bajas de levodopa de una sola formulación, y la mayor frecuencia de síntomas no motores sin adecuado diagnostico ni tratamiento, especialmente ansiedad, depresión y deterioro cognitivo. Estos hallazgos pueden ser explicados en parte por la limitación en el acceso a otras formulaciones de levodopa y a terapias avanzadas como la estimulación cerebral profunda. Estos hallazgos resaltan la oportunidad de optimizar el tratamiento de síntomas motores y no motores con terapias farmacológicas y no farmacológicas, enfocados en mejorar la calidad de vida de los pacientes con EP que viven en la región Arequipa, idealmente como parte de programas de tratamiento integral que incluyan terapia física, psicoterapia, y planes de neuro rehabilitación funcional.
